# Mapping global risk levels of *Bemisia tabaci* in areas of suitability for open field tomato cultivation under current and future climates

**DOI:** 10.1371/journal.pone.0198925

**Published:** 2018-06-14

**Authors:** Rodrigo Soares Ramos, Lalit Kumar, Farzin Shabani, Marcelo Coutinho Picanço

**Affiliations:** 1 Department of Entomology, Universidade Federal de Viçosa, Viçosa, Minas Gerais, Brazil; 2 Ecosystem Management, School of Environmental and Rural Science, University of New England (UNE), Armidale, New South Wales, Australia; 3 Global Ecology, College of Science & Engineering, Flinders University, Adelaide, South Australia, Australia; Sichuan University, CHINA

## Abstract

The whitefly, *Bemisia tabaci*, is a major threat to tomato *Solanum lycopersicum* and ranks as one of the world’s 100 most invasive pests. This is the first study of *B*. *tabaci* (Biotype B and Q) global distribution, focusing on risk levels of this invasive pest, in areas projected to be suitable for open field *S*. *lycopersicum* cultivation under climate change. This study aims to identify levels of risk of invasive *B*. *tabaci* for areas of suitability for open field *S*. *lycopersicum* cultivation for the present, 2050 and 2070 using MaxEnt and the Global Climate Model, HadGEM2_ES under RCP45. Our results show that 5% of areas optimal for open field *S*. *lycopersicum* cultivation are currently at high risk of *B*. *tabaci*. Among the optimal areas for *S*. *lycopersicum*, the projections for 2050 compared to the current time showed an extension of 180% in areas under high risk, and a shortening of 67 and 27% in areas under medium and low risk of *B*. *tabaci*, respectively, while projections for 2070 showed an extension of 164, and a shortening of 49 and 64% under high, medium and low risk, respectively. The basis of these projections is that predicted temperature increases could affect the pest, which has great adaptability to different climate conditions, but could also impose limitations on the growth of *S*. *lycopersicum*. These results may be used in designing strategies to prevent the introduction and establishment of *B*. *tabaci* for open-field tomato crops, and assist the implementation of pest management programs.

## 1. Introduction

Plant pest expansion increased in the last century, mainly due to international travel and the trading of plants around the world [1]. A pest species may be distributed in different regions of the planet, introduced either by natural or anthropic dispersion. After introduction, the pest species may establish and cause negative impact to local hosting ecosystem and economy. Many factors may influence expansion, such as the availability of hosts and appropriate climatic conditions [2,3,4,5].

Climate is a major factor impacting on the distribution and abundance of arthropod species [6,7]. Studies of climate effects on pests and host species have advanced significantly in recent years and attained greater relevance, in terms of impact on distribution, physiology, phenology, genetics and behaviors of many invasive species [8]. It is predicted that climate change will have a great impact on agricultural crops such as date palm [9], maize [10], wheat and cotton [11], rice [12], as well as insect populations, both in natural ecosystems and agroecosystems [13].

The term ‘climate change’ refers to both global-scale and regional climate alterations over time. It is an important concept in organism distribution studies, especially in the case of insects, which are classified as ectotherms [14]. As particular regions become warmer, colder, wetter or dryer, they may become more or less suitable for specific pests and hosts. For example, a mean temperature increase hastens the maturing of many insect species, consequently affecting life cycle length, reproductive capacity and the degree of mobility [8]. Thus, the distribution of a species is primarily dependent on climate, which generally defines its geographical distribution.

A species distribution model (SDM) is a tool widely used in understanding the effects of climate change on a species, as well as planning further expansion of agricultural species and potential risks this may entail [15,16]. Such models offer the most efficient techniques for simulation of future climates under a variety of climate scenarios. They offer a means to study the projected impact of climate change on pest distribution. The model generates categories of climate suitability and matches these to geographical regions, whether or not the species occurs there currently. Such research, based on modeling, can predict the distribution and abundance of pests, in addition to elucidating ecological interactions and abiotic factors affecting the natural mortality.

The whitefly *Bemisia tabaci* (Gennadius) (Hemiptera: Aleyrodidae) has been shown to be one of the most invasive and devastating insect pests of agricultural and horticultural crops, causing enormous economic losses worldwide [17,18]. The species is sap-sucking, highly generalist and widely distributed, making it a pest of great significance in many tropical countries. *B*. *tabaci* is ranked globally as one of the top 100 most invasive pests and has the ability to colonize on more than 600 plant species [18,19]. Among the species of plants colonized by the *B*. *tabaci*, the tomato *Solanum lycopersicum* cultivated in many countries and one of the most valuable vegetable crops globally, is one of the crops worst affected by the pest [20,21,22]. The damage caused by *B*. *tabaci* can be either through direct feeding (phloem sap-sucking), or due to the injection of toxins and transmission of over 150 plant viruses, mainly of the genus Begomovirus (Family: Geminiviridae), which is significant in tomato crops [23,24,25]. Damage caused by this insect pest may lead to mortality of the plants, with losses of up to 100% of production [23,26,27,28]. Thus, *B*. *tabaci* is a major problem in tomato crops. Cases of productivity losses due to the presence of the pest in open field tomato cultivation are more and more frequent, and concern is even greater where the insect is already present but the viruses (i.e Geminivirus) have not yet been reported. The combination can make tomato production unfeasible in many production fields around the world.

*Bemisia tabaci* is considered a species complex containing more than 30 morphological indistinguishable cryptic species [29], between them there are two most invasive and destructive species, which are the Middle East-Asia minor I, and the Mediterranean species, also referred as Biotype B and Q, respectively [17,29,30]. *B*. *tabaci* may fly long distances and acquire new niches, mostly by international trade. It is well known that the biotypes of *B*. *tabaci* are very dynamic and acquire new properties, and may replace another within a few years (e.g. Biotype B replacing A in the USA, and Q replacing B in the Middle and Far East). For this reason, it is important to consider these two biotypes in the model because they are showing to be similar in many aspects beyond climate requirements, and they are the ones, so far, that have strongly suppressed other biotypes especially when they reach places where only wild biotypes are present. Hence these two biotypes are considered most invasive and important to tomato crops [17], and for being similar on their climate niches [31], we selected the occurrences of these two biotypes B and Q for undertaking the modelling in this study.

Despite the impact of the whitefly and the large body of research on the species, the potential impact of climatic change on the global distribution of *B*. *tabaci* in agricultural crops remains understudied, particularly in tropical regions such as Brazil. So far there are only two modelling studies, one assessing the risk presented by *B*. *tabaci* in Europe [32] and another a case study in Bundaberg, Australia as adaptive pest management for horticulture under climate change [33]. Reviewing related research, our study appears to be unprecedented in its focus on risk levels of invasive pest of *B*. *tabaci*, in areas suitable for *S*. *lycopersicum* cultivation under climate change. In this research, we modeled the risk of *B*. *tabaci* in *S*. *lycopersicum* cultivation for the present and years 2050 and 2070. Thereafter, we overlaid these results onto predicted *S*. *lycopersicum* future distributions, to establish categories of highest, medium and lowest risk for areas highly conducive to the cultivation of *S*. *lycopersicum*. Predicting geographical distributions of *B*. *tabaci* facilitates the development of models and consequently the concentration of efforts in regions with higher risk of invasion or establishment. Furthermore, analysis of the potential impact of climate change on areas of suitability for *S*. *lycopersicum* open-field cultivation, for both present and future are essential for the continued success of producing an economically viable crop and the design of more efficient strategies for controlling whitefly in open-field tomato farming, in terms of *B*. *tabaci*’s global ranking as a threat and the difficulties in attempting to control it [17,18,34], particularly in tomatoes [35]. Though *B*. *tabaci* is present on all continents, it is not yet established worldwide in open field scenarios [36].

## 2. Material and methods

### 2.1. Occurrence data

Occurrence data for both species examined in our research was collected from GBIF and other references (for *B*. *tabaci*, Biotype B and Q): 878 locations from GBIF and 84 from other literature and for *S*. *lycopersicum*: 186 occurrences from literature). We confirmed that all locations were open field, as opposed to glasshouses. Eventually, we selected 627 occurrences for *B*. *tabaci* and retained the 186 occurrences for *S*. *lycopersicum*. The *B*. *tabaci* records were reduced to 421 and *S*. *lycopersicum* to 177 after spatial filtering in spThin, an R package for minimizing spatial autocorrelation [37]. This technique retains as many localities as possible and outperforms alternative methods [38]. All occurrence data points were >10 km apart after filtering [38,39]. This distance ensures that each cell has only a single occurrence point.

### 2.2. Scenario and model

Potential distributions of pest and host were modelled using the HadGEM2_ES GCM under the RCP45, for the years 2050 and 2070.

RCP45 was developed by the GCAM (Global Change Assessment Model) modeling team at the Pacific Northwest National Laboratory's Joint Global Change Research Institute (JGCRI) (http://www.globalchange.umd.edu/models/gcam/) The scenario is structured on the core assumption that the global irradiative force will be stabilized by 2100 through the introduction of greenhouse gas limiting technology [40]. A secondary assumption is that forest land cover will be extended and crop and grazing lands reduced to increase carbon storage. RCP45 was selected on the basis that it sets out to describe the minimum aggregated impact of climate change [41].

HadGEM2-ES is a product of the Hadley Centre Global Environmental Model associated cycle of the fifth phase of the CMIP5 (http://www.ipcc.ch/report/ar5/wg1/) [42]. The model incorporates dynamic data on the impact of greenhouse gas emissions, aerosols, solar irradiance, ozone and other pollutants on vegetation, ocean biology and atmospheric chemistry [43]. With a CO_2_ doubling rate of approximately 4.68°C, it ranks near the top of the CMIP5 range for climate sensitivity [43,44]. The coupling of the atmospheric and ocean models simulates the uptake and retention of carbon dioxide according to ocean depth more realistically than its predecessors [45].

### 2.3. Environmental data layers

We initially considered nineteen bioclimatic parameter variables (Tables 1 and 2), from the WorldClim dataset [46] (http://www.worldclim.org/), at 2.5min spatial resolution (~5Km). This is a high quality resolution, sufficient to support climatic variables at global scale [2,47]. Average temperature was based on monthly climate data for minimum, mean, and maximum temperature and precipitation on data covering 1960–1990. Other parameters were drawn from seasonal variables and climatic extreme indices [46].

**Table 1:** Environmental variables considered in *B*. *tabaci* (Biotype B and Q) niche models, and average percent contribution of environmental variables in the *B*. *tabaci* (Biotype B and Q) distribution model; values were averaged across 10 replicate runs. General statistics were calculated using all occurrences (n = 627). (Min = minimum, Max = maximum, and SD = standard deviation).

Variable	Percent contribution	Permutation importance	Min.	Max.	Mean	SD
**Annual mean temperature (bio1; °C)**	75.1	65.4	8.0	28.7	23.8	3.8
**Precipitation seasonality (CV) (bio15)**	7.2	6.4	0	160	89.4	30.3
**Mean annual precipitation (bio12; mm)**	6.5	8.5	0	3516	667	588.8
**Precipitation of driest month (bio14; mm)**	6.1	3.6	0	113	9.7	17.4
**Mean diurnal range in temperature (bio2; °C)**	2.7	7.8	6.0	18.9	13	2.7
**Temperature annual range (bio7; °C)**	2.4	8.3	8.5	40.7	28.4	9.7
Isothermality (bio3)	-	-	24	91	49.4	14.5
Temperature seasonality (SD x 100) (bio4)	-	-	251	10871	5199	2761
Maximum temperature of warmest month (bio5; °C)	-	-	22.8	46.3	37.4	5.6
Minimum temperature of coldest month (bio6; °C)	-	-	-9.9	22.8	8.93	6.4
Mean temperature of wettest quarter (bio8; °C)	-	-	6.2	34.5	27.2	6.4
Mean temperature of driest quarter (bio9; °C)	-	-	-2.6	36.1	21.7	4.8
Mean temperature of warmest quarter (bio10; °C)	-	-	16	36.1	29.9	4.5
Mean temperature of coldest quarter (bio11; °C)	-	-	-2.6	36.1	16.7	5.4
Precipitation of wettest month (bio13; mm)	-	-	0	815	140	113.7
Precipitation of wettest quarter (bio16; mm)	-	-	0	1875	348	293
Precipitation of driest quarter (bio17; mm)	-	-	0	381	41	62.6
Precipitation of warmest quarter (bio18; mm)	-	-	0	1219	189	178
Precipitation of coldest quarter (bio19; mm)	-	-	0	1865	118	174.5

Bold font indicates variables in the final model. Source of data: WorldClim (http://www.worldclim.org/bioclim; Hijmans et al., 2005).

**Table 2:** Environmental variables considered in *S*. *lycopersicum* niche models, and average percent contribution of environmental variables in the *S*. *lycopersicum* distribution model; values were averaged across 10 replicate runs. General statistics were calculated using all occurrences (n = 627). (Min = minimum, Max = maximum, and SD = standard deviation).

Variable	Percent contribution	Permutation importance	Min.	Max.	Mean	SD
**Annual mean temperature (bio1; °C)**	58.5	57.4	8.6	28.8	20.1	4.3
**Temperature annual range (bio7; °C)**	25.9	25.1	9.7	37.7	20.1	6.6
**Mean diurnal range in temperature (bio2; °C)**	8.5	7.8	7.1	17.0	11.3	19.3
**Mean annual precipitation (bio12; mm)**	5.9	6.7	3	3200	1039.6	598.6
**Precipitation seasonality (CV) (bio15)**	0.7	1.9	11	145	65.7	29.4
**Precipitation of driest month (bio14; mm)**	0.5	1.0	0	120	22.8	26.8
Isothermality (bio3)	-	-	24	91	59.9	15.4
Temperature seasonality (SD x 100) (bio4)	-	-	163	9490	2907.9	2204.8
Maximum temperature of warmest month (bio5; °C)	-	-	18.7	41.2	30.2	3.7
Minimum temperature of coldest month (bio6; °C)	-	-	-8.9	22.9	10.1	6.5
Mean temperature of wettest quarter (bio8; °C)	-	-	5.6	29.1	20.5	5.6
Mean temperature of driest quarter (bio9; °C)	-	-	-3.2	31.5	19.9	5.4
Mean temperature of warmest quarter (bio10; °C)	-	-	13.3	32.7	23.7	3.5
Mean temperature of coldest quarter (bio11; °C)	-	-	-3.2	27.9	16.3	6.3
Precipitation of wettest month (bio13; mm)	-	-	1	727	180.5	112.6
Precipitation of wettest quarter (bio16; mm)	-	-	3	1806	471.6	286
Precipitation of driest quarter (bio17; mm)	-	-	0	447	85.4	93.2
Precipitation of warmest quarter (bio18; mm)	-	-	0	1388	282.6	208.8
Precipitation of coldest quarter (bio19; mm)	-	-	0	1097	201.3	217.6

Bold font indicates variables in the final model. Source of data: WorldClim (http://www.worldclim.org/bioclim; Hijmans et al., 2005.

SDMtools in ARCGIS software was used to remove variables with high correlation, such that only one variable from a group with high correlation was included (Pearson correlation coefficient, r ≥|0.75|) (S1 Table). Values exceeding 0.75 are described by Kumar et al. (2014) as relatively strong for variable selection [48]. Ultimately the inclusion of a variable was based on realistic biological relevance to both *B*. *tabaci* and *S*. *lycopersicum*, and six bioclimatic variables were finally selected (Table 1; S1 Table).

### 2.4. Model development and validation

Global potential distributions of *B*. *tabaci* and *S*. *lycopersicum* were obtained from the maximum entropy based model or MaxEnt algorithm version 3.3.3k [49]. MaxEnt constitutes a machine learning method that forecasts the probability distribution based on maximum entropy [49]. MaxEnt requires only a small sampling of data on the presence of a species and the background data [48,50,51,52]. The program most suited our research which had only presence data available for pest and host [49]. MaxEnt generates a suitability index ranging between 0 for unsuitable and 1 for optimum suitability. 50,000 background points were randomly selected for each species representing areas of current occurrence. A sampling bias was generated in that the data was collected unsampled from external sources. This was generated using a kernel density estimate in SDMToolbox [53]. The bias surface offsets sampling intensity and potential sampling bias [54].

To optimize the model for both *B*. *tabaci* and *S*. *lycopersicum*, we made adjustments to the MaxEnt default settings for certain combinations of feature types, as well as the regularization multiplier (RM). [48,54,55]. Initially, we combined sets of linear [L], quadratic [Q], product [P], threshold [T], and hinge [H] features (Tables 3 and 4) with the RM to control the number of parameters and thus the model complexity [55,56]. RM values of 1.0; 1.5 and 2.0 were used for both species (Tables 3 and 4).

**Table 3:** Summary of performance statistics of *B*. *tabaci* (Biotype B and Q) MaxEnt models. The best model is highlighted in bold.

ModelRank	Variables	MaxEnt settings		Test AUC_cv_ (±SD)	OR	
		Features	RM		0%	10%
**1**	**bio1,bio2,bio7, bio12, bio14, bio15**	**LQPH**	**1.0**	**0.900 ± 0.017**	**0.0071**	**0.1086**
2	Same as above	LQPTH	2.0	0.896 ± 0.017	0.0048	0.1108
3	Same as above	LQH	1.0	0.889 ± 0.017	0.0096	0.1108
4	Same as above	LQP	2.0	0.886 ± 0.017	0.0024	0.113
5	Same as above	LQP	1.0	0.886 ± 0.017	0.0048	0.1134
6	Same as above	LQPH	2.0	0.896 ± 0.017	0.0024	0.1155
7	Same as above	LQPH	1.5	0.897 ± 0.016	0.0048	0.1155
8	Same as above	LH	2.0	0.884 ± 0.017	0.0024	0.1158
9	Same as above	LQH	2.0	0.882 ± 0.017	0.0024	0.1204
10	Same as above	LH	1.0	0.892 ± 0.017	0.0024	0.1205
11	Same as above	LQPTH	1.5	0.896 ± 0.017	0.0048	0.1226
12	Same as above	LQH	1.5	0.884 ± 0.017	0.0048	0.1227

**Note:** Variables’ full names (see table 1). L, Q, P, T and H are linear, quadratic, product, threshold and hinge features, respectively. RM is regularization multiplier, and SD is standard deviation. OR is test omission rate. Test AUC_cv_ is MaxEnt 10-fold cross-validation Area Under the ROC curve.

**Table 4:** Summary of performance statistics of *S*. *lycopersicum* MaxEnt models. The best model is highlighted in bold.

ModelRank	Variables	MaxEnt settings		Test AUC_cv_ (±SD)	OR	
		Features	RM		0%	10%
**1**	**bio1,bio2,bio7, bio12, bio14, bio15**	**LH**	**1.0**	**0.904 ± 0.023**	**0.0059**	**0.1016**
2	Same as above	LQP	2.0	0.902 ± 0.024	0.0059	0.1033
3	Same as above	LQPT	2.0	0.904 ± 0.024	0.0059	0.1075
4	Same as above	LH	2.0	0.902 ± 0.024	0.0173	0.1078
5	Same as above	LQP	1.0	0.901 ± 0.024	0.0110	0.1127
6	Same as above	LQH	2.0	0.901 ± 0.025	0.0118	0.1127
7	Same as above	LQH	1.5	0.903 ± 0.024	0.0056	0.1131
8	Same as above	LQPH	1.5	0.903 ± 0.023	0.0059	0.1150
9	Same as above	LQPH	2.0	0.898 ± 0.024	0.0110	0.1196
10	Same as above	LQPTH	1.5	0.906 ± 0.024	0.0167	0.1199
11	Same as above	LH	1.5	0.904 ± 0.025	0.0114	0.1203
12	Same as above	LQP	1.5	0.899 ± 0.024	0.0056	0.1206

**Note:** Variables’ full names (see Table 1). L, Q, P, T and H are linear, quadratic, product, threshold and hinge features, respectively. RM is regularization multiplier, and SD is standard deviation. OR is test omission rate. Test AUC_cv_ is MaxEnt 10-fold cross-validation Area Under the ROC curve.

An RM below 1 is restrictive and inappropriate for global predictions, while an RM greater than 1 produces a broader potential distribution [49].

We used the MaxEnt ‘fade-by-clamping’ option to eliminate extrapolations outside the environmental range [57]. The predictive contribution of environmental variables was estimated using the ‘jackknife’ technique. MaxEnt generates response curves and we chose only those representing relationships between probabilities of presence for each species, in terms of each environmental predictor. All response curves were evaluated on the basis of sound biological logic and those failing this test were eliminated from further evaluations.

Test sensitivities of 0% and 10% training Omission Rates (OR) [52,58] and the AUC_cv_ (area under the receiver operating characteristic [ROC] curve) were used to compare performance of the models [59]. To calculate these, a 10-fold cross-validation was run in MaxEnt. AUC_cv_ was also used for discriminating presence from background data. An AUC_cv_ value of 0.5 indicates that predictions do not exceed random; values below 0.5 are below random; 0.5–0.7 represents poor performance; 0.7–0.9 represents satisfactory to moderate performance; and values above 0.9 rate as high performance [60]. In the case of Omission Rate, the anticipated value at 0% training OR is 0 and at 10% it is 0.10; poor performance is indicated when the value exceeds the anticipated rate [38]. We ranked our models on 10% training OR, 0% training OR, and AUC_cv_ [55,58,61].

ArcGIS 10.3.1 software was used to extract the risk levels for *B*. *tabaci* invasion of areas suitable for open field cultivation of tomatoes from the MaxEnt outputs.

### 2.5. Determining *B*. *tabaci* risk levels

Maximum Test Sensitivity Plus Specificity (MTSPS) threshold was chosen to ascertain from projected future distributions of *B*. *tabaci* and *S*. *lycopersicum* which areas optimal for open field cultivation of *S*. *lycopersicum* would be at risk for *B*. *tabaci* invasion, according to the categories of highest, medium and lowest risk. For overlaying, ‘optimal conditions’ were defined as areas of medium or high suitability. Projections for both species, as well as their overlaying to ascertain level of *B*. *tabaci* risk in areas of optimal risk, were made using the four suitability classes of unsuitable, low, medium and high.

## 3. Results

**Fig 1:**
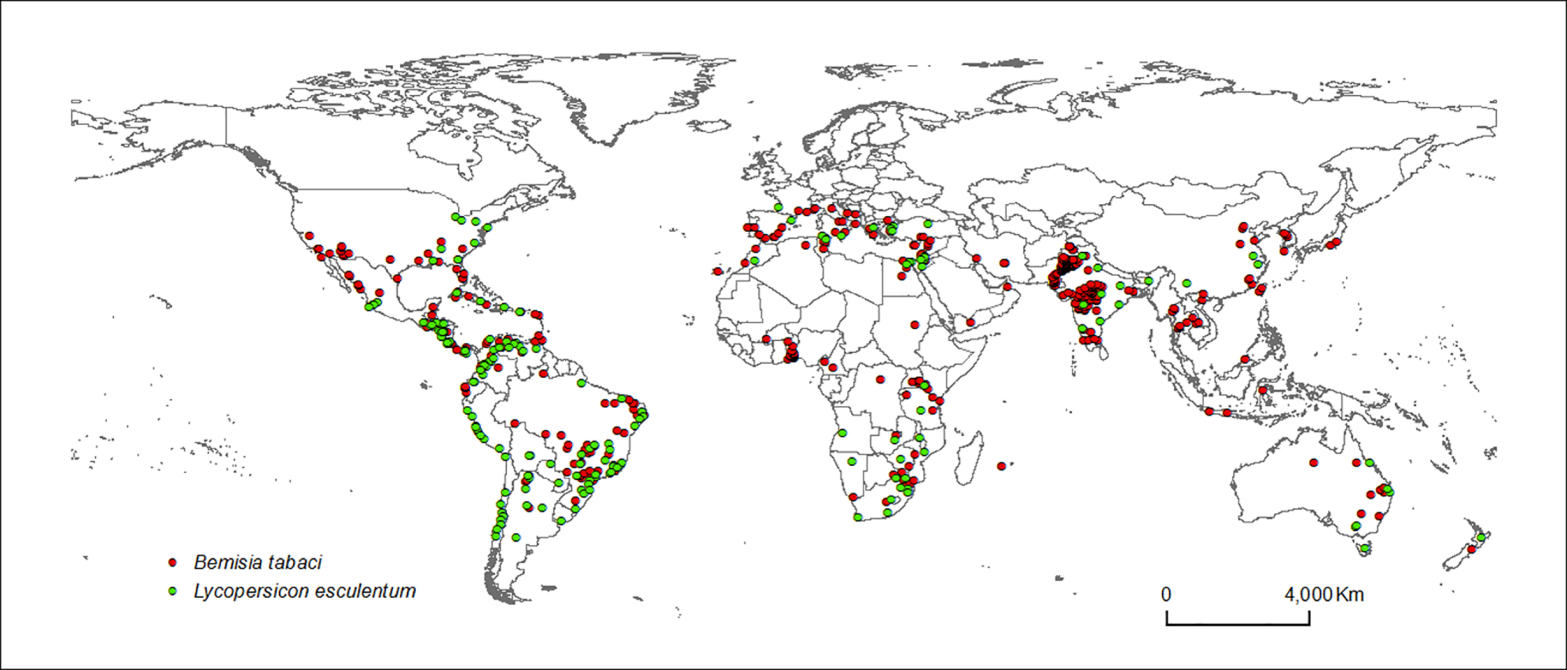
Global known occurrences of *B*. *tabaci* (Biotype B and Q) in open field (red dots), and *S*. *lycopersicum* in open field (green dots).

The pest species *B*. *tabaci* (Biotype B and Q) and host open-field *S*. *lycopersicum* are distributed globally (Fig 1). Climatic variables most contributory to *B*. *tabaci* distribution were annual mean temperature (bio1; °C), precipitation seasonality (CV) (bio15), mean annual precipitation (bio12; mm), precipitation of driest month (bio14; mm), mean diurnal range in temperature (bio2; °C) and temperature annual range (bio7; °C) (Table 1), and *S*. *lycopersicum* (Table 2). Based on observed occurrences, *B*. *tabaci* (Biotype B and Q) occurs in areas with mean annual temperature of 23.8°C, and mean annual precipitation between 0–3516 mm (Table 1) and *S*. *lycopersicum* occurs in areas with mean annual temperature of 20.1°C, and mean annual precipitation from 3000–3200 mm (Table 2).

The performance of all MaxEnt models used to determine the two species potential distributions exceeded random, using AUC_cv_ test values higher than 0.5 (Tables 3 and 4). Based on 10-fold cross validation, mean AUC_cv_ values varied from 0.882–0.900 for *B*. *tabaci* (Table 3) and 0.898–0.904 for *S*. *lycopersicum* (Table 4). These models also had low test omission rates, with values at 0% training OR, varying from 0.0024–0.0096, and 0.1086–0.1227 at 10% for *B*. *tabaci* (Table 3). For *S*. *lycopersicum*, values at 0% training OR varied from 0.0056–0.0173, and from 0.1016–0.1206 at 10% (Table 4). The best model for *B*. *tabaci* included a combination of six environmental variables, Linear, Quadratic, Product and Hinge (LQPH) features, regularization multiplier = 1.0, produced the best model for *B*. *tabaci* and exhibited the lowest omission rate at 10% and 0% (Table 3). Similarly for *S*. *lycopersicum*, the best model had six environmental variables, Linear and Hinge (LH) features, regularization multiplier = 1, and had lowest omission rate at 10% and 0% (Table 4).

**Fig 2:**
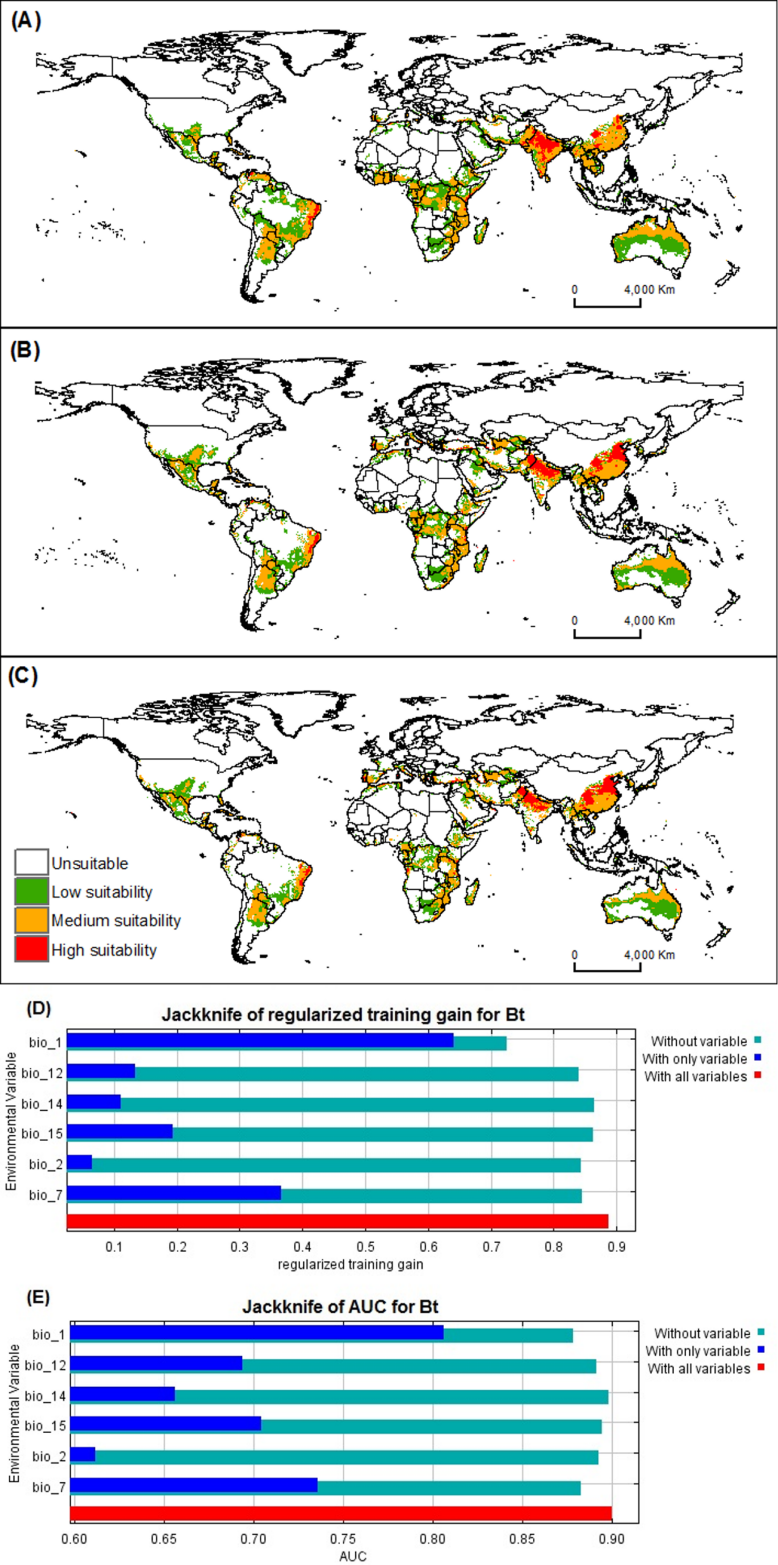
Habitat suitability under current and future climatic conditions of *B*. *tabaci* (Biotype B and Q). Maps (A) current global distribution using MaxEnt model, (B) 2050, (C) 2070. Relative importance of the environmental variables based on the JackKnife test (D) Regularized training gain and (E) AUC in *B*. *tabaci* model.

**Fig 3:**
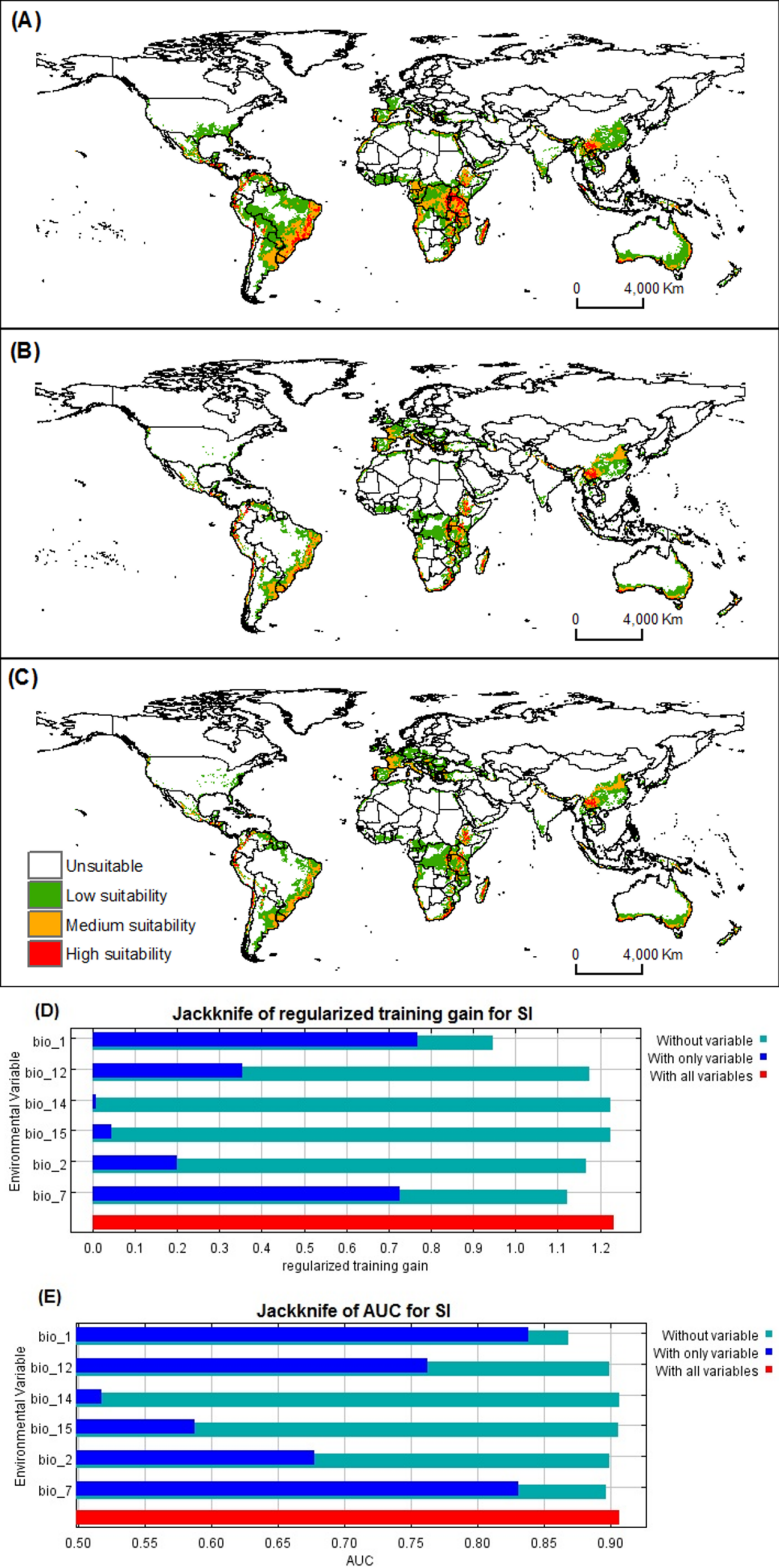
Habitat suitability under current and future climatic conditions of open-field *S*. *lycopersicum* cultivation. Maps (A) current global distribution using MaxEnt model, (B) 2050, and (C) 2070. Relative importance of the environmental variables based on the JackKnife test (D) Regularized training gain and (E) AUC in *S*. *lycopersicum* model.

MaxEnt predictions of the best *B*. *tabaci* model closely matched current known distributions (Figs 1 and 2). Highly suitable areas were predicted in South America, Africa, Europe, Asia and Oceania. The *S*. *lycopersicum* model also displayed agreement between known occurrences and projections, globally (Figs 1 and 3).

**Fig 4:**
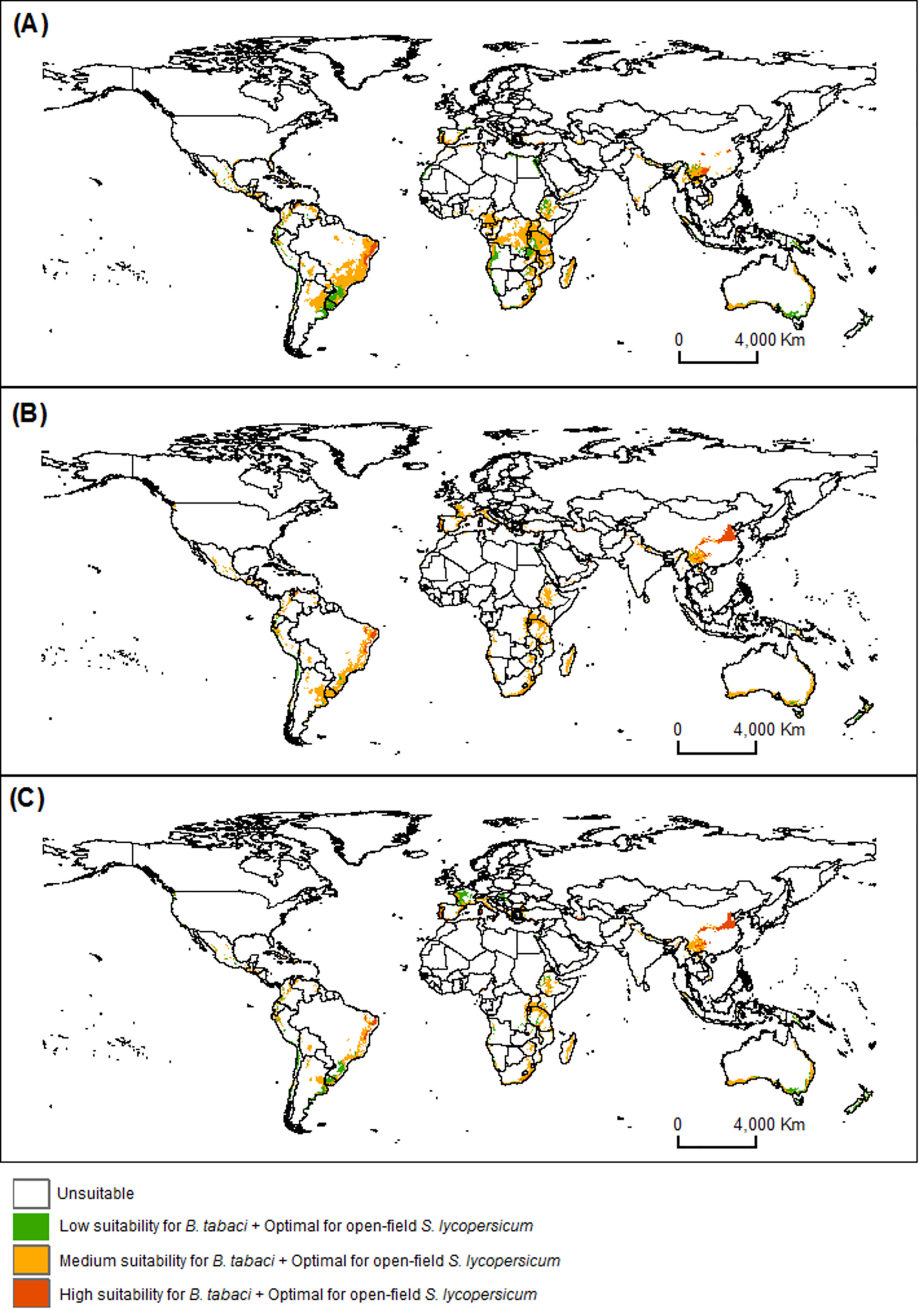
Habitat suitability under current and future climatic conditions in optimal areas for open-field *S*. *lycopersicum* cultivation with three suitability levels of *B*. *tabaci* (Biotype B and Q) using MaxEnt model. Maps (A) current time, (B) 2050, and (C) 2070.

The current and projected climate results for *B*. *tabaci* risk levels in areas with optimal conditions (medium and high suitability) for *S*. *lycopersicum* open-field cultivations for 2050 and 2070 are shown in Fig 4. Results indicated that 5% of optimal areas are currently at high *B*. *tabaci* risk. Currently, much of South and Central America has a low, medium or high risk levels for *B*. *tabaci* in areas with optimal conditions for open-field tomato. On the North American continent, Mexico and the USA states of California and Florida exhibit low, medium or high risk. In all the above regions, *B*. *tabaci* is already present. In Europe, Africa, Asia and Oceania all three risk categories for *B*. *tabaci* can be discerned. Many sites in Europe and southern China which are at high risk for *B*. *tabaci* in optimal conditions for tomato open-field require close attention (Fig 4A).

However, a decrease in areas with low or moderate levels of *B*. *tabaci* is projected for 2050 and 2070, but an increase of areas with high risk for *B*. *tabaci* (Fig 4B and 4C). Among the optimal areas for *S*. *lycopersicum*, the projections for 2050 compared to the current time showed an extension of 180% in areas under high risk, and a shortening of 67 and 27% in areas under medium and low risk of *B*. *tabaci*, respectively. By 2070, the projections indicate an extension of 164, and a shortening of 49 and 64% under high, medium and low risk, respectively. According to projected scenarios for 2050 and 2070 the risks levels for *B*. *tabaci* in China will increase from the east to the centre of the country when compared to the current levels (Fig 4A, 4B and 4C). In Europe, the current *B*. *tabaci* risk level is moderate and high in southern regions but will increase to more moderately suitable for *B*. *tabaci* in regions with optimal climatic conditions for open-field *S*. *lycopersicum*.

In large areas such as South America (for example Brazil), the current risk levels of *B*. *tabaci* are moderate and high (Fig 4A). However, most optimal regions already produce open-field tomatoes. Future predictions show large reductions of areas with *B*. *tabaci* risk levels due to the reduction of climatic conditions suitable for both species, but mostly for the host (Fig 4A, 4B and 4C).

**Fig 5:**
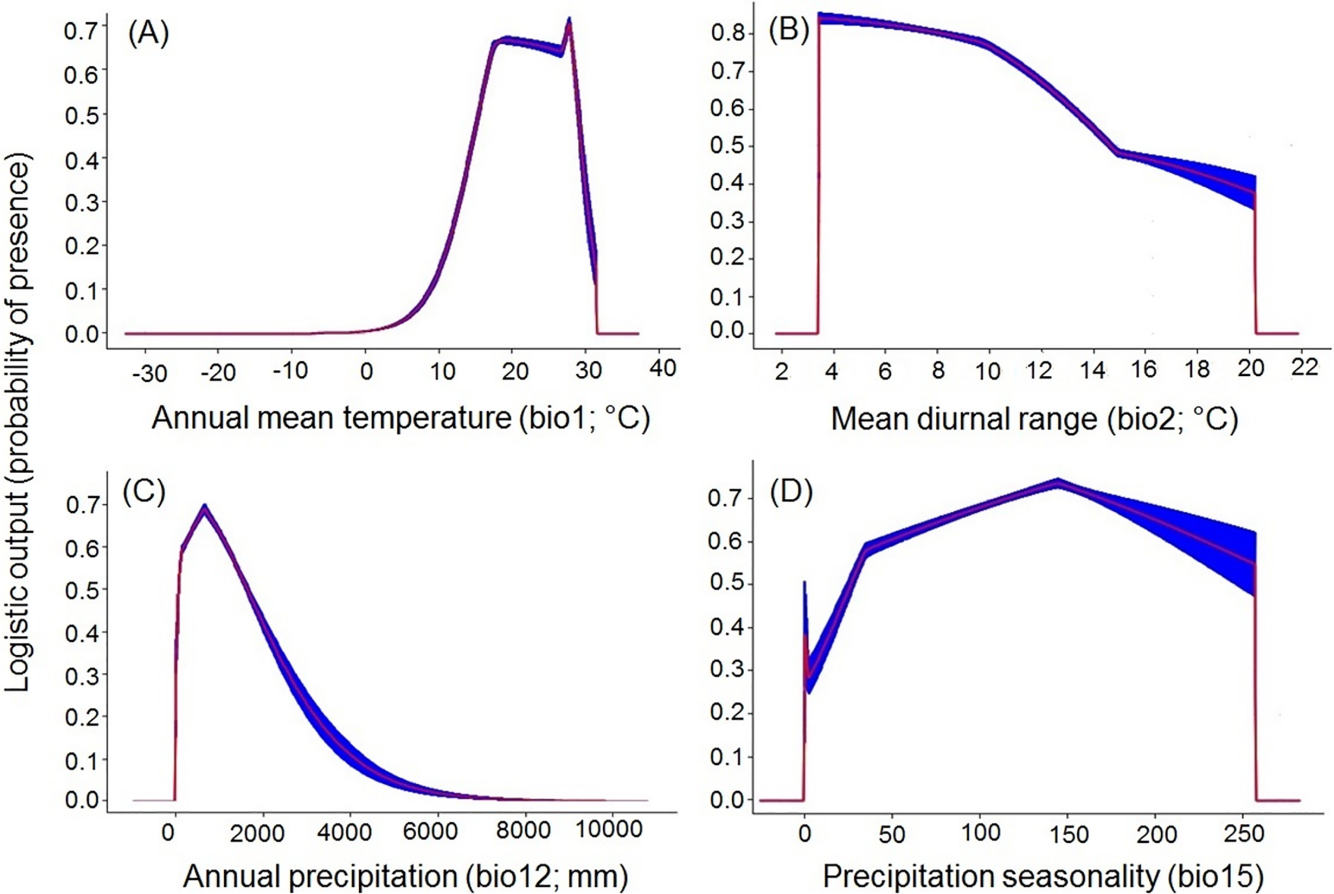
Response curves of the best predictors of *B*. *tabaci* (Biotype B and Q) in the best model. (A) Annual mean temperature (bio1; °C), (B) Mean diurnal range (Mean of monthly (max temp–min temp)) (bio2), (C) Annual precipitation (bio12; mm), and (D) Precipitation seasonality (Coefficient of variation, bio15).

**Fig 6:**
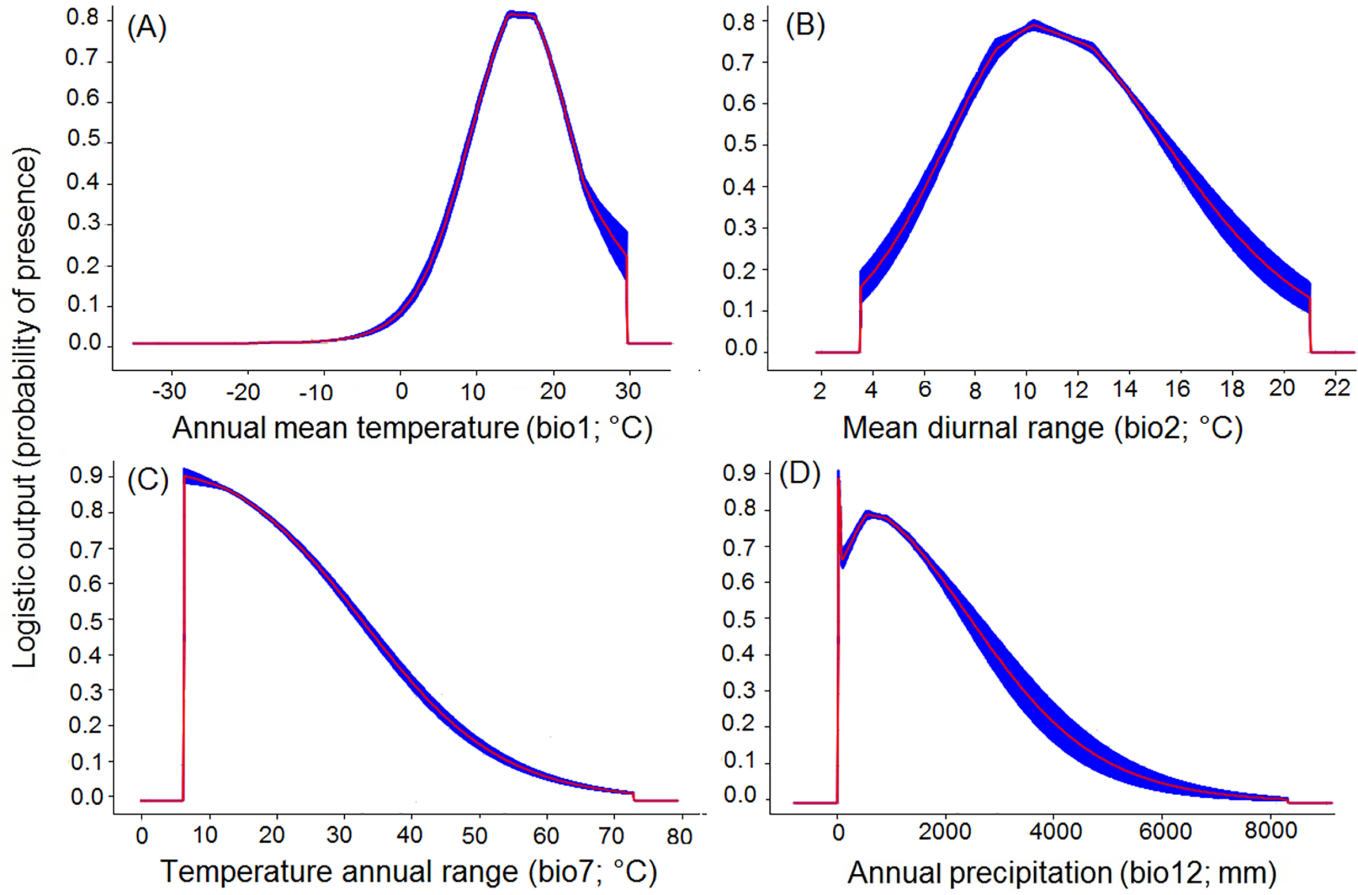
Response curves of the best predictors of *S*. *lycopersicum* in the best model. (A) Annual mean temperature (bio1; °C), (B) Mean diurnal range (Mean of monthly (max temp–min temp)) (bio2), (C) Temperature annual range (bio7; °C), and (D) Annual precipitation (bio12; mm).

The Jackknife test of variable importance indicated that mean annual temperature had the most impact on both species models (*B*. *tabaci* and *S*. *lycopersicum*) (Fig 2D and 2E and Fig 3D and 3E). The highest probability for *B*. *tabaci* presence exits in areas with annual mean temperatures of 23–24°C (Fig 5A), and 18–20°C for *S*. *lycopersicum* (Fig 6A). The probability of *B*. *tabaci* presence is higher in areas of low mean diurnal temperature range (Fig 5B) and higher in areas of medium mean diurnal temperature range for *S*. *lycopersicum* (Fig 6B). The probability for the presence of both species is higher in areas with low precipitation, decreasing with an increase in precipitation (Figs 5C and 6D), and when the precipitation seasonality is 130–150 mm for *B*. *tabaci* (Fig 5D), and temperature annual range is low for *S*. *lycopersicum* (Fig 6C).

## 4. Discussion

*B*. *tabaci* is distributed worldwide (Fig 1) and has caused many losses in tomato crops over the past 20 years in North, Central and South America, Africa (West, Central and South), Asia (India and China) and Mediterranean Europe countries [62,63,64,65,66].

All models presented in this study show great degree of reliability. Both *B*. *tabaci* and *S*. *lycopersicum* models produced 90% (AUC) agreement with the current modeled global climate. Cross-validation indicated that all models performed much better than would be expected at random, and had a high validation statistic. The high percentage of accordance with the distribution of both species highlights the consistency of these models. Thus, the model outputs and their overlaying may be considered reliable as a foundation of research on *B*. *tabaci* occurrence and risk in areas suitable for open-field tomato, globally.

While *B*. *tabaci* and *S*. *lycopersicum* occurrence has been reported for regions on all continents (Fig 1), the combination is more probable in tropical regions with high mean annual temperatures (Tables 1 and 2 and Figs 5A and 6A), including areas with wide variations of daily temperatures (Figs 5B and 6B), and a wider range in precipitation (Tables 1 and 2 and Figs 5C and 6D). These important characteristics confirm that *B*. *tabaci* has great potential for invasion [67,68,69] and that *S*. *lycopersicum* may be produced in open field conditions in several areas in the world. However, in certain regions where *B*. *tabaci* has already been reported in glasshouses, it has not been reported as causing problems in the open field. This is probably because climatic conditions are unsuitable for *B*. *tabaci* growth, development and therefore the establishment of the species in these areas.

Although *B*. *tabaci* can establish across a wide thermal range, the species can be affected by global temperature changes (Fig 2). Extreme temperatures (either high or low) may affect the development of the species. Furthermore, pests are dependent on the survival of the host. Thus, tomato crops may well suffer from climate change, which will have an impact on *B*. *tabaci* [70] (Fig 3).

If the increases in temperature predicted by the HadGEM2-ES climate model do occur, this may affect not only the pest but impose limitations on *S*. *lycopersicum* growth. Vegetable species generally show great sensitivity under extreme environmental conditions, particularly temperatures, high or low temperatures [71]. This was confirmed by the results of the future models (2050 and 2070), in which greater reductions in susceptibility are predicted in *S*. *lycopersicum* globally, than in *B*. *tabaci* (Figs 2 and 3).

The current climate modeling showed predominantly medium and high risk in South, Central and North America for *B*. *tabaci* in areas with optimal climate conditions for growing open-field tomato (Fig 4A). Europe, Africa, Asia and Oceania displayed all three categories of risk of *B*. *tabaci*; with close attention for many sites in Europe, southern China (Asia) and Brazil (South America), which are at high risk for *B*. *tabaci* in optimal conditions for open-field tomato.

While the majority of studies on the impact of climate change on organisms predict increases in invasive species, the converse may be seen for *B*. *tabaci* in open field tomato in some regions. After the overlaying of the current models and future projections in areas with optimal conditions for open field tomato, we observed that in some regions with current high risk, *B*. *tabaci* risk will decrease in the years 2050 and 2070, mainly in tropical countries of South America and Africa. The main reason related to the predicted increase of temperature in the world, which will mainly affect the host. Conversely, in China (from east to central areas), and Mediterranean Europe (e.g. France, Italy and Spain) a great increase in susceptibility to *B*. *tabaci* is predicted in regions with optimal climatic conditions for open field tomato crops. In some areas along the coastlines of Brazil (south, southeast and northeast) and Australia (south and east), the same levels of susceptibility to, or risk of, the pest are maintained (Fig 4).

At high temperatures, the current B and Q *B*. *tabaci* biotypes exhibit lower fertility than at temperate climates [72]. However, new emerging biotypes that are much more resistant to heat may be selected during climatic warming. This is something that we cannot include in our model because we do not know how often new biotypes in the species will emerge, and if the resistance will occur at the same speed that climate change. The biological factors such as generation time and how climate change may influence the appearance of new biotypes are characteristics that are still not possible to be included as parameters in the construction of models via MaxEnt.

Mean annual temperature (bio1) was one of the most important variables associated with the distribution of *B*. *tabaci* and *S*. *lycopersicum* (Tables 1 and 2, Figs 2D, 2E, 3D and 3E respectively). Many studies have shown that mean annual temperature is the major variable contributing to distributions of species [73,74,75,76]. The models predicted a greater probability of presence of *B*. *tabaci* in temperatures around 23–24°C and around 18–20°C for *S*. *lycopersicum*. This may be the major reason that *B*. *tabaci* occurrence has been so high in areas having a mean temperature close to the optimal requirement for growth. However, a combination of major climate factors with other variables, such as a higher number of hosts, might be related to the success of the *B*. *tabaci* distributions [77,78]. Consequently, the decreased *B*. *tabaci* risk levels in some areas may be due to a reduction of climatic suitability factors for both species.

Tomato production becomes unviable in places where temperatures reach values above 40°C. There are many studies that show that high temperature causes stress in tomato plants (for the most common tomato cultivars) such as reduced fruit set, reproductive number, pollen production and pollen viability [70,79,80]. *S*. *lycopersicum* shows a wide climatic tolerance and is grown in both tropical and temperate regions around the world, and thus high or low temperatures can impact negatively on this species [81]. As shown by our model, the mean annual temperature is an extremely important parameter that determines the distribution (occurrence and establishment) of the species. Although there are varieties more adapted for high temperature sites (e.g. in the Middle East) they can still yield at high temperatures, mostly due the manipulation of the environment (nethouses and glasshouses), using ventilation or air-conditioning. Obviously, with the advance in genetic studies, new cultivars could be introgressed by breeding and selection into current genotypes when needed to cope with increasing temperatures. The same has happened in countries where the mean temperatures are very low and tomato production is only possible due to the temperature control possible in the greenhouse (where it is possible to maintain higher temperatures than the external environment). Therefore, the evolution and emergence of adapted varieties in high temperature environments may succeed as long as productivity is not compromised. Even with the advent of current plant genotypes resistant to high temperatures, this is not yet a reality that extends to the entire planet and we do not know if those varieties will support high temperatures and if they would be an alternative to overcome the barriers to climate change. In our model, we used the occurrence points of tomato plants of current and most commonly used cultivars. However, the production of new cultivars resistant to high temperatures can make tomato production feasible in places where air temperatures are quite high. This is one of the uncertainties that we could not include in the MaxEnt configuration for building our model.

Our overlaying results should contribute to warning agricultural authorities in many locations to employ management strategies to prevent a decreased viability for open-field tomato. In areas of high *B*. *tabaci* risk and optimal conditions for open-field *S*. *lycopersicum*, both whitefly and tomato are already present. It would still be logical to introduce preventive measures for the spread of toxins and viruses into areas in which these have not yet been reported. Strategies such as inspection of seeding plant trade and phytosanitary regulations would be valid in locations with high and medium risk of *B*. *tabaci*, to lessen the risk.

Despite the inherent uncertainty of correlative niche models such as MaxEnt in regard to the quality of occurrence data, resolution of spatial data layers, sampling bias, species characteristics, and spatial autocorrelation [54,76,82,83,84], MaxEnt software has a great user interface, making the modeling process easier. MaxEnt does offer options for certain adjustments, which can improve the quality of specific models [54,61,76]. The adjustments used in our study were in the selection of feature types, value of regularization multiplies, selection of background points and extent [49]. Utmost care was devoted to model calibration to obtain model results adhering to the current occurrence of both species studied. The quality of the models can be seen in the biological validity of the response curves and strong validation results (Tables 2 and 3; Figs 2D and 2E; 3D and 3E; 5 and 6).

Our models were based only on climate parameters, executed using the currently available global broad-scale climate data, and thus only show broad-scale shifts. Only open field occurrence data for both *B*. *tabaci* and *S*. *lycopersicum* were taken into account.

It should be noted that the predictions of suitable areas for tomato production were established based on the current climatic thresholds from current commercial cultivars. However, the production of new cultivars resistant to high temperatures, the production of tomatoes in protected crops with climatic control or the production of tomato in times of mild temperature in very hot places can enable the future production of tomato in other places. Although tomato cultivation is still mainly cultivated in the open field, the current trend in tomato cultivation is increasingly favouring nethouses (50-mesh nets to protect from insects) or closed glasshouses. In both cases, proper ventilation or air-conditioning can control inside temperatures. These systems control the microclimate of crops and modelling studies can not take this into account. For this reason, the problems with *B*. *tabaci* in protected and semi-protected environments may be diverse in several locations around the world but not pointed out by our model.

Although it is well known that the *B*. *tabaci* is already widely distributed and has other host species, modeling other hosts may have predictions that differ from our study, on the basis of specific host sensitivity to climatic changes. In this study, we did not take into account potential genetic progress as *B*. *tabaci* has shown high adaptability and numerous biotypes. Therefore, the results of these models can be used in other studies, including non-climatic factors such as differences of the existing biotypes, other pest-plant interactions, natural enemies, pest resistance, dispersal and adaptations.

Our study indicates that climate change may impact on the geographical distributions of the pest *B*. *tabaci* and the host *S*. *lycopersicum*. Our study provides important information on the risk of *B*. *tabaci* for open-field tomato crops using the MaxEnt model. Considering both species together (*B*. *tabaci* and *S*. *lycopersicum*), it seems large areas with optimal conditions for *S*. *lycopersicum* under current climate are already at medium and high risk of *B*. *tabaci*, areas with high risk of *B*. *tabaci* will increase and areas with medium risk will decrease in the future (2050 and 2070). The future projections in areas with optimal conditions for open field tomato shows that some regions (e.g. Brazil), where *B*. *tabaci* currently shows medium and high risk will become less favorable (risks will decrease) in the years 2050 and 2070. Conversely in some places such as China (from east to central areas), and Europe (e.g. France, Italy and Spain) projections show large increases in susceptibility to *B*. *tabaci* in regions with optimal climatic conditions for tomato crops in open field. The main reason for this is related to the predicted increase of temperature in the world, which may affect not only the pest, but also the host. Our results can be used in designing strategies to prevent the introduction and establishment of *B*. *tabaci* in areas still *B*. *tabaci* free, such as Finland, Sweden, Republic of Ireland and the UK, as well as implementing pest management programs in areas of current occurrence, particularly at sites under high risk.

## Supporting information

S1 Table Cross-correlation (Pearson correlation coefficient, r) among environmental variables. (Models: *B*. *tabaci* (Biotype B and Q) and *S*. *lycopersicum*).
(ZIP)
