# Supplementary material for: Mapping global risk levels of Bemisia tabaci in areas of suitability for open field tomato cultivation under current and future climates
Source: PLoS One. 2018 Jun 14;13(6):e0198925. doi: 10.1371/journal.pone.0198925 (PMC6002045; doi:10.1371/journal.pone.0198925)
Supplement: S1 Table — (Models: B. tabaci (Biotype B and Q) and S. lycopersicum). (ZIP) [file pone.0198925.s001.zip › S1 Table.doc]

**S1 Table .** Cross-correlation (Pearson correlation coefficient, r) among environmental variables. (Models: *B. tabaci* (Biotype B and Q) and *S. lycopersicum*).

|  | **bio1** | bio2 | bio3 | bio4 | bio5 | bio6 | bio7 | bio8 | bio9 | bio10 | bio11 | bio12 | bio13 | bio14 | bio15 | bio16 | bio17 | bio18 | bio19 |
| --- | --- | --- | --- | --- | --- | --- | --- | --- | --- | --- | --- | --- | --- | --- | --- | --- | --- | --- | --- |
| **bio2** | 0.521 |  |  |  |  |  |  |  |  |  |  |  |  |  |  |  |  |  |  |
| bio3 | 0.839 | 0.387 |  |  |  |  |  |  |  |  |  |  |  |  |  |  |  |  |  |
| bio4 | -0.833 | -0.210 | -0.891 |  |  |  |  |  |  |  |  |  |  |  |  |  |  |  |  |
| bio5 | 0.896 | 0.707 | 0.613 | -0.513 |  |  |  |  |  |  |  |  |  |  |  |  |  |  |  |
| bio6 | 0.968 | 0.355 | 0.888 | -0.936 | 0.764 |  |  |  |  |  |  |  |  |  |  |  |  |  |  |
| **bio7** | -0.730 | 0.015 | -0.826 | 0.971 | -0.358 | -0.876 |  |  |  |  |  |  |  |  |  |  |  |  |  |
| bio8 | 0.812 | 0.533 | 0.638 | -0.501 | 0.845 | 0.704 | -0.387 |  |  |  |  |  |  |  |  |  |  |  |  |
| bio9 | 0.938 | 0.439 | 0.808 | -0.861 | 0.792 | 0.949 | -0.781 | 0.609 |  |  |  |  |  |  |  |  |  |  |  |
| bio10 | 0.935 | 0.621 | 0.660 | -0.585 | 0.988 | 0.825 | -0.455 | 0.865 | 0.831 |  |  |  |  |  |  |  |  |  |  |
| bio11 | 0.980 | 0.425 | 0.891 | -0.926 | 0.795 | 0.996 | -0.847 | 0.731 | 0.950 | 0.848 |  |  |  |  |  |  |  |  |  |
| **bio12** | 0.378 | -0.245 | 0.565 | -0.554 | 0.119 | 0.486 | -0.615 | 0.252 | 0.375 | 0.200 | 0.454 |  |  |  |  |  |  |  |  |
| bio13 | 0.455 | -0.104 | 0.581 | -0.567 | 0.233 | 0.527 | -0.589 | 0.370 | 0.413 | 0.303 | 0.511 | 0.896 |  |  |  |  |  |  |  |
| **bio14** | 0.055 | -0.375 | 0.221 | -0.245 | -0.139 | 0.167 | -0.346 | -0.066 | 0.107 | -0.075 | 0.123 | 0.709 | 0.392 |  |  |  |  |  |  |
| **bio15** | 0.367 | 0.514 | 0.282 | -0.190 | 0.429 | 0.269 | -0.069 | 0.449 | 0.260 | 0.412 | 0.317 | -0.173 | 0.138 | -0.517 |  |  |  |  |  |
| bio16 | 0.445 | -0.124 | 0.581 | -0.569 | 0.215 | 0.522 | -0.595 | 0.352 | 0.408 | 0.286 | 0.504 | 0.922 | 0.993 | 0.429 | 0.094 |  |  |  |  |
| bio17 | 0.083 | -0.372 | 0.255 | -0.275 | -0.121 | 0.197 | -0.375 | -0.044 | 0.135 | -0.054 | 0.153 | 0.744 | 0.429 | 0.994 | -0.515 | 0.466 |  |  |  |
| bio18 | 0.224 | -0.202 | 0.353 | -0.337 | 0.026 | 0.282 | -0.389 | 0.254 | 0.153 | 0.100 | 0.265 | 0.797 | 0.743 | 0.558 | -0.104 | 0.761 | 0.581 |  |  |
| bio19 | 0.249 | -0.242 | 0.431 | -0.405 | 0.055 | 0.357 | -0.475 | 0.074 | 0.307 | 0.112 | 0.316 | 0.754 | 0.584 | 0.671 | -0.273 | 0.610 | 0.698 | 0.374 |  |

Bold font indicates variables in the final model.
